# Genetic Analysis of Patients with Chronic Thromboembolic Pulmonary Hypertension (CTEPH): A Single-Center Observational Study

**DOI:** 10.3390/genes16111336

**Published:** 2025-11-06

**Authors:** Zsuzsanna Bereczky, Gábor Kolodzey, Sarolta Borsos, László Balogh, Petra Erzsébet Biró, Éva Molnár, Katalin Rázsó, Andrea Péter, Judit Barta, Tibor Szűk

**Affiliations:** 1 Division of Clinical Laboratory Science, Department of Laboratory Medicine, Faculty of Medicine, University of Debrecen, 4032 Debrecen, Hungary; borsos.sarolta@med.unideb.hu (S.B.); biropetrae@gmail.com (P.E.B.); molnare@med.unideb.hu (É.M.); 2 Department of Cardiology and Cardiac Surgery, Faculty of Medicine, University of Debrecen, 4032 Debrecen, Hungary; kolodzey.gabor@med.unideb.hu (G.K.); laszlo.balogh@med.unideb.hu (L.B.); peter.andrea@med.unideb.hu (A.P.); barta.judit@med.unideb.hu (J.B.); tszuk@med.unideb.hu (T.S.); 3 Department of Internal Medicine, Faculty of Medicine, University of Debrecen, 4032 Debrecen, Hungary; krazso@med.unideb.hu

**Keywords:** chronic thromboembolic pulmonary hypertension, CTEPH, pulmonary embolism, next-generation sequencing, thrombosis and hemostasis, coagulation, fibrinolysis, vascular disorders, variant detection, mutation

## Abstract

**Background/Objectives**: Chronic thromboembolic pulmonary hypertension (CTEPH) is a rare disease, in which multiple genetic and environmental factors may contribute. This study aimed to identify potential genetic determinants in patients with CTEPH and to compare their occurrence to a control group, which included patients with pulmonary embolism who had not developed CTEPH. **Methods**: Tier 1 and 2 genes related to coagulation, fibrinolysis and platelet disorders—as recommended by the International Society on Thrombosis and Haemostasis—and genes associated with vascular conditions were analyzed in n = 15 patients with CTEPH and n = 17 controls using next-generation sequencing. Non-synonymous, rare variants were collected and interpreted. **Results**: As expected, no single gene or variant was consistently present among CTEPH patients. Instead, individuals carried different mutations and combinations of variants. We identified several variants that were not found in the control group. Candidate variants were detected in *F12*, *F13A1*, *F13B*, *F5*, *KNG1*, *SERPIND1*, *THBD*, *ADAMTS13*, *VWF*, *STIM1*, *ETV6*, *THPO*, *MPL*, *SERPINA1*, *ENG*, *RASA1*, *ACVRL1*, *GDF2*, *NFE2*, *SOX17* and *RNF213.* We did not detect exclusive variants in *FGA*, *CPB2*, and *BMPR2* although they were suggested as candidates in previous studies. Elevated factor VIII and von Willebrand factor in CTEPH could not be explained by mutations in *VWF* and *F8*. **Conclusions**: Our study supports the hypothesis of heterogeneous genetic background in CTEPH, involving multiple pathways such as coagulation, altered fibrinolysis and impaired angiogenesis. These results provide a basis for more detailed investigations into specific genes and variants potentially associated with CTEPH in larger cohorts.

## 1. Introduction

Pulmonary embolism (PE) is a life-threatening thromboembolic disorder and a leading cause of cardiovascular morbidity and mortality worldwide [1,2]. The incidence of acute PE is estimated to be 39–115 cases per 100,000 people annually, with significant variation based on geographical and population-specific factors. Although thrombolytic therapy and anticoagulation are effective in resolving most PE cases, a subset of patients experiences incomplete thrombus resolution, leading to chronic thromboembolic pulmonary hypertension (CTEPH) [3,4]. This progressive disorder, which occurs in approximately 2–4% of PE survivors, is associated with increased pulmonary vascular resistance, right ventricular dysfunction, and ultimately right heart failure if left untreated [5,6,7,8]. The diagnosis of chronic thromboembolic pulmonary hypertension (CTEPH) is established in patients who present with persistent pulmonary hypertension (mean pulmonary artery pressure ≥ 20 mmHg, pulmonary artery wedge pressure ≤ 15 mmHg, and pulmonary vascular resistance > 2 Wood units) confirmed by right heart catheterization, in combination with imaging evidence of chronic thromboembolic obstruction, such as mismatched perfusion defects on ventilation/perfusion (V/Q) scanning, or organized thromboembolic lesions visualized on pulmonary angiography, CT pulmonary angiography (CTPA), or MR angiography [9]. Diagnosis of CTEPH requires that these findings persist despite effective anticoagulation therapy for at least three months in order to exclude subacute or resolving thromboembolic disease.

CTEPH is a complex and multifactorial condition, with acquired and genetic risk factors contributing to its pathogenesis [10,11,12,13]. Acquired risk factors include previous deep vein thrombosis (DVT), malignancy, splenectomy, functional asplenia, chronic inflammatory diseases and antiphospholipid syndrome. However, not all individuals with these risk factors will suffer from CTEPH, suggesting that genetic predisposition may also play a critical role in determining disease susceptibility. The presence of genetic susceptibility is also supported by familial clustering studies [14]. As thrombophilic risk factors were presented in a high proportion of individuals suffering from CTEPH, research into genes related to thrombotic disorders has been conducted. Several candidate genes have been identified in relation to thrombus formation and fibrinolysis, each of which may contribute to the persistence of thrombi and subsequent vascular remodeling, leading to the pathologic condition of CTEPH [15]. Results of genetic studies executed so far, however, are rather heterogeneous. Although some studies have suggested that variations in *SERPINC1*, a gene encoding antithrombin (AT), may increase the risk of PE and CTEPH due to impaired anticoagulant activity, classical hereditary thrombophilia risk factors (i.e., causative mutations in *SERPINC1, PROC, PROS1* and presence of factor V Leiden and prothrombin 20210A) seemed not to be independent risk factors of CTEPH [16,17]. Mutations in *FGA* and *FGB*, which encode alpha and beta chains of fibrinogen, respectively, have been associated with altered clot structure and resistance to fibrinolysis, potentially predisposing individuals to persistent thrombotic occlusions and the development of CTEPH [18,19,20]. Platelet abnormalities, especially their hyperreactivity in the background of CTEPH, are also plausible hypotheses [21]. Impairment in fibrinolysis would be a logical explanation for why thrombi are not dissolved after PE, leading to organization of pulmonary thrombi; however, no clear conclusions can be drawn from the studies executed so far [22,23].

As microvascular remodeling is suggested to have a major role in CTEPH development [24,25], mutations in genes related to vascular development and signaling, being linked to abnormal vascular remodeling and impaired endothelial barrier function, are also targets of research. This hypothesis is strengthened by the observation that many patients with a definitive CTEPH diagnosis have no history of symptomatic PE; however, they may have had a subclinical thromboembolic event [26].

It is still unknown why CTEPH develops only in a minority of patients after PE and which factors contribute to each other, leading to this unique condition. Development of CTEPH after PE is still unpredictable and we are not able to select cases with a high CTEPH risk, although it would be of utmost importance from the point of view of patient management. Research into this field is difficult due to the several potentially contributing pathways and the low number of CTEPH cases even at large clinical centers. Several risk factors seen in CTEPH overlap with PE and venous thromboembolism, making finding predisposing factors for CTEPH even more difficult [27,28]. To better separate risk factors of CTEPH from those of thrombosis, it would be straightforward to compare the findings to a group of PE patients without the development of CTEPH.

High-throughput genetic methods, like next-generation sequencing (NGS), are powerful tools for identifying genetic variants associated with a certain phenotype. By focusing on protein-coding regions of the genome, whole-exome sequencing (WES) enables the detection of mutations that may disrupt key physiological pathways related to thrombosis and vascular homeostasis.

Our aim was to identify genes and their variations with a plausible role in the development of CTEPH, by using next-generation sequencing (NGS). This study aimed to analyze the differences between PE patients who develop CTEPH and those who do not in order to identify and interpret variants in association with thrombus formation, persistence, impaired fibrinolysis, and endothelial dysfunction.

## 2. Materials and Methods

### 2.1. Study Design and Population

An open-label, non-randomized, prospective observational study was conducted at the Department of Cardiology, University of Debrecen. Following approval of the study protocol, patients were prospectively enrolled after providing written informed consent. Prior to enrollment, all participants received detailed information regarding the investigational nature of the study, including potential risks and anticipated benefits.

Patients included in the study (n = 15) were diagnosed with CTEPH and met the established diagnostic criteria (please see above). The suspicion of pulmonary hypertension was raised based on echocardiographic findings and was subsequently confirmed by right heart catheterization using a Swan–Ganz catheter and pulmonary angioplasty. All patients enrolled in the study underwent balloon pulmonary angioplasty (BPA). Patient enrollment commenced in 2022, with follow-up continuing through 2025. Comprehensive demographic and clinical data—including vital status, hospital admissions, comorbid conditions, treatment history, and follow-up outcomes—were extracted from hospital records. The control group consisted of 17 patients with a history of pulmonary embolism in whom echocardiography performed 3 to 6 months after the acute event excluded the development of pulmonary hypertension.

### 2.2. Laboratory Testing of CTEPH and PE Patients

Blood samples were collected in 3.2% Na-citrate anticoagulated tubes (Greiner, Kremsmunster, Austria). Laboratory testing included screening tests of coagulation and fibrinogen detection by the Clauss method on BCS-XP coagulometer (Siemens, Marburg, Germany). Coagulation factor VIII was measured by the chromogenic method (Siemens FVIII Chromogenic Assay), von Willebrand factor antigen was measured by the Innovance VWF assay (Siemens), and plasminogen and alpha2-plasmin inhibitor were detected by Berichrom plasminogen and Berichrom alpha2-antiplasmin, respectively (Siemens). Protein C (PC), protein S (PS) and antithrombin (AT) were measured by Berichrom Protein C, Innovance free PS Ag and Innovance Antithrombin, respectively, on a BCS-XP coagulometer. Lupus anticoagulant testing was executed according to the current guidelines by the ISTH, using diluted Russel’s viper venom time and lupus anticoagulant-sensitive APTT (Werfen, Milan, Italy and Diagnostica Stago, Asnieres, France, respectively) [29]. Anticardiolipin and anti-beta2 glycoprotein I IgG and IgM antibodies were detected by chemiluminescent immunoassays on a Bioflash analyzer (Werfen) [30]. Coagulation FXIII activity was determined by the modified optimized kinetic spectrophotometric ammonia-release assay on a Sysmex CS2500 coagulometer (Siemens) by using Technoclone FXIII reagent (Technoclone, Vienna, Austria). Markers of a pro-thrombotic state, thrombin–antithrombin complex (TAT) and prothrombin fragment 1+2 (PF1+2), were detected by ELISA (Enzygnost TAT micro and Enzygnost F1+2, respectively; both were purchased from Siemens). D-dimer was measured by HemosIL D-dimer HS500 on an ACL-TOP coagulometer (Werfen). PAI-1 antigen was measured by Technozym PAI-1 antigen ELISA (Technoclone), and tPA was detected by Human Tissue Type Plasminogen Activator ELISA kit (Abcam, Cambridge, UK). TFPI concentration was measured by ELISA from Invitrogen (Thermo Fisher, Waltham, MA, USA).

### 2.3. Genetic Testing of CTEPH and PE Patients

DNA was isolated from peripheral blood leukocytes by QIAmp DNA Blood Mini kit (Qiagen, Hilden, Germany). After isolation, the purity of DNA was checked by NanoDrop 2000 (Thermo Fisher Scientific, Waltham, MA, USA). DNA concentration was determined by Qubit^®^ dsDNA HS Assay Kits on a Qubit Fluorometer (Thermo Fisher), then DNA was diluted to yield 200 ng DNA in 30 μL. Library preparation for clinical exome sequencing was executed using the Clinical exome solution v3 kit (SOPHiA GENETICS, Lausanne, Switzerland) according to the manufacturer’s instructions. Quality control analysis of the library pools was performed by Agilent Fragment Analyzer capillary electrophoresis (Agilent, Santa Clara, CA, USA). Next-generation sequencing (NGS) was performed on an Illumina NextSeq 500 instrument (Illumina, San Diego, CA, USA) with the NextSeq 500/550 Mid Output Kit v2.5 (300 Cycles).

Factor V Leiden (rs6025) and prothrombin 20210A (rs1799963) polymorphisms were detected by a LightCycler 480 instrument (Roche, Basel, Switzerland) by using real-time PCR followed by melting curve analysis with in-house-designed primers (TIB^®^ MOLBIOL, Berlin, Germany) and probes (Roche). Primers were as follows: FII forward: 5′-CCG CTG GTA TCA AAT GGG-3′; FII reverse: 5′-CCA CTA GTA TTA CTG GCT CTT CCT G-3′; FV forward: 5′-TAATCTGTAAGAGCAGA XT CC-3′, where X = BODYPY630/650 NHS ester; FV reverse: 5′-TGTTATCACACTGGTGCTAA-3′. Probes were as follows: FII anchor probe: 5′-X TCC CAG TGC TAT TCA TGG GC Y-3′, where X = BODYPY630/650, Y = 3Phos; FII sensor probe: 5′-CTC AGC GAG CCT CAA TG X-3′, where X = 6FAM; and FV sensor probe: 5′-AATACCTGTATTCCTCGCCTGTC X-3′, where X = 6FAM. The PCR procedure was executed using the Genotyping Master kit (Roche).

### 2.4. Data Analysis and Statistics

Continuous variables are reported as either mean ± standard deviation (SD) or median with range, depending on the underlying distribution. Categorical variables are presented as absolute frequencies and percentages. The normality of data distributions was assessed using the Kolmogorov–Smirnov test. Student’s *t*-test or the Mann–Whitney U-test was carried out in order to compare the values between two groups. In the case of categorical variables, chi-square test was used. A two-tailed *p*-value of <0.05 was considered indicative of statistical significance. All analyses were performed using IBM SPSS Statistics, version 29.

Bioinformatical analysis of NGS data was executed by SOPHiA DDM software v6.4, and annotation was performed using the hg38 reference genome. Two virtual gene panels were generated: The first one included Tier 1 genes associated with coagulation, fibrinolysis and platelets, as recommended recently by the International Society on Thrombosis and Haemostasis (ISTH, https://www.isth.org/page/GinTh_GeneLists (accessed on 25 July 2025)). The second group of genes was selected based on their association with vascular conditions (please see below). Variants captured by the software were described according to the American College of Medical Genetics and Genomics (ACMG) recommendations and were classified into pathogenic, likely pathogenic, VUS, likely benign and benign according to its built-in algorithm, which uses data from different genetic databases and in silico prediction software (PolyPhen2, SIFT, MutPred, Mutation Taster, v6.4) [31]. Selected variants were also checked manually in the available genetic databases, Human Gene Mutation Database (HGMD, http://www.hgmd.cf.ac.uk), Online Mendelian Inheritance in Men (OMIM, https://www.omim.org/) and ClinVar (https://www.ncbi.nlm.nih.gov/clinvar/ (accessed between 1 August 2025 and 25 September 2025)), for classification. Allele frequency data of variants were obtained from the gnomAD and 1000 genomes databases (https://gnomad.broadinstitute.org/ and https://www.internationalgenome.org/).

### 2.5. Ethical Statement

The study was approved by the Regional Scientific and Ethical Committee of the University of Debrecen, Clinical Center, as well as by the National Scientific and Research Ethics Committee of Hungary (Approval No: RKEB/IKEB: 6153-2022). Written informed consent was obtained from all participants prior to inclusion, in accordance with the principles of the Declaration of Helsinki.

## 3. Results

### 3.1. Characteristics of the Patients with CTEPH

The CTEPH cohort included both male and female patients (10 males, 5 females), all of whom were of white (Caucasian) ethnicity. Their demographic and laboratory characteristics are demonstrated in Table 1. The mean age at diagnosis showed a wide distribution, and their body mass index showed a wide range from normal weight to severe obesity, with the highest BMI value of 34.29. At the time of enrollment, most patients (n = 10) were classified as New York Heart Association (NYHA) functional class II or III, indicative of moderate functional limitation, while a smaller proportion (n = 3) presented in class IV. The mean distance achieved on the 6 min walk test (6MWT) was 336.1 ± 164.7 m (range: 42–616 m), reflecting substantial variability in exercise capacity. The average baseline level of N-terminal pro-brain natriuretic peptide (NT-proBNP) was 2694.3 ± 2407.2 pg/mL (range: 82–7133), consistent with variable degrees of right ventricular (RV) dysfunction. Left ventricular function was preserved in all patients.

Systolic pulmonary artery pressures, as measured by echocardiography and Swan–Ganz catheterization, averaged 77.2 ± 23.5 mmHg and 72.9 ± 22.6 mmHg, respectively. Right heart catheterization further confirmed elevated pulmonary vascular resistance (mean PVR: 720.3 ± 373.5 dyn · s · cm^−5^) and increased mean pulmonary artery pressure (mPAP: 44.0 ± 9.1 mmHg). Pulmonary capillary wedge pressure (PCWP) and right atrial pressure (RAP) were 11.7 ± 3.3 mmHg and 7.6 ± 4.2 mmHg, respectively. Cardiac output (CO) and cardiac index (CI) were reduced (median CO: 3.82, range 2.72–8.53 L/min; CI: 2.03, range 1.66–4.04 L/min/m^2^), consistent with compromised right ventricular performance.

Medical history revealed a high thromboembolic burden, as six patients had a documented history of pulmonary embolism (PE), and two were found to have established inherited thrombophilia (one patient homozygous for the prothrombin gene 20210A mutation, rs1799963, and one patient heterozygous for factor V Leiden, rs6025, Supplementary Table S1). No AT, PC and PS deficiencies were registered. One patient had lupus anticoagulant and another one had moderate elevation of anti-beta 2 glycoprotein I IgG (133.2 CU). An elevated factor VIII (FVIII) level above 200 IU/dL was observed in one patient, while elevated von Willebrand factor antigen (vWF:Ag) above 200 IU/dL was measured in five patients. No patient had a history of splenectomy. Electrocardiographic findings commonly included right bundle branch block (RBBB) and patterns of RV strain; atrial fibrillation was observed in a minority of patients. There were no cases of provoked PE in our study population—neither in the CTEPH group nor in the control group—with no transient risk factors such as surgery, trauma, or immobilization identified. No patients with hemoglobinopathies, including sickle cell disease (SCD), were found in our cohort. During follow-up, six patients died, including three from non-cardiovascular causes—COVID-19, pneumonia, and septic shock.

### 3.2. Characteristics of Patients with PE Without the Development of CTEPH

The baseline demographic and anthropometric characteristics are shown in Table 1. The mean body mass index ranged from normal weight to severe obesity (BMI 44.6). In this group, three patients stopped anticoagulation therapy 6 months after the acute event, while three patients were on long-term rivaroxaban, nine patients were on apixaban and two patients were on dabigatran at the time of investigation. Among PE patients, there were two FV Leiden heterozygotes and two patients carried the prothrombin 20210A allele in heterozygous form. No classical AT, PC and PS deficiencies were registered; however, one patient was a heterozygous carrier of the PS Heerlen polymorphism (rs121918472, c.1501T > C, p.Ser501Pro) with a free PS antigen level of 63%, which is considered a mild risk factor for thrombosis [32]. One patient had lupus anticoagulant and none of the PE patients had elevated antiphospholipid antibody values. An elevated vWF:Ag level exceeding 200 IU/dL was detected in one patient; her FVIII activity was 190 IU/dL.

### 3.3. Comparison of Laboratory Parameters Between CTEPH Patients and PE Patients

Among parameters reflecting coagulation, CTEPH patients had significantly elevated FVIII and vWF:Ag levels as compared to controls (Table 1). Fibrinogen concentration did not differ between the two groups. D-dimer was also not different; however, parameters reflecting pro-thrombotic states (TAT complex and PF1+2) were significantly higher in patients with PE without the development of CTEPH, suggesting a continuously higher level of coagulation activation, which was not so pronounced in CTEPH patients. TFPI levels were not different between the two groups. By investigating factors involved in fibrinolysis, plasminogen and alpha2-PI levels were significantly lower in CTEPH individuals; however, on the contrary, tPA was significantly higher. There was no difference in PAI-1 and FXIII levels. These results suggest—although the sample size is very low—that the lower plasminogen level is associated with higher tPA activity and with lower alpha2-PI activity in CTEPH patients, resulting in an altered balance in fibrinolysis. This observation, however, does not allow us to draw a clinically meaningful conclusion, since none of these laboratory parameters fell out of the corresponding reference intervals and no extreme values were detected. Elevation in FVIII and vWF:Ag levels in CTEPH was more pronounced, suggesting the role of endothelial activation in this disease.

### 3.4. Investigation of Genes Associated with Hemostasis and Thrombosis in CTEPH and PE Patients

**Figure 1:**
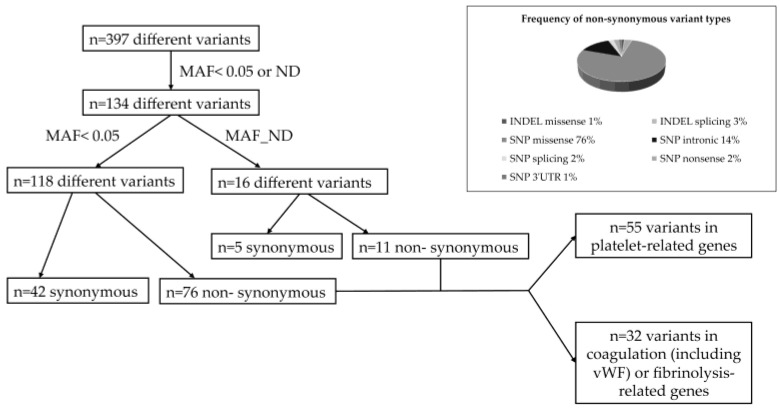
Algorithm of variant detection in CTEPH patients. Frequency of different non-synonymous variants. MAF, minor allele frequency; ND, non-determined; vWF, von Willebrand factor; INDEL, insertion or deletion; SNP, single-nucleotide substitution.

First, we established a virtual gene panel including Tier 1 genes as recommended by the SSC Subcommittee on OMICS in Thrombosis and Hemostasis of the ISTH, (https://www.isth.org/page/GinTh_GeneLists, accessed on 2 November 2025). This gene list currently contains 109 genes associated with coagulation, fibrinolysis and platelet disorders. By this search, n = 397 different variants were found within CTEPH patients. After excluding common variants with higher allele frequency values than 0.05, as based on 1000genomes and/or gnomAD data, n = 134 different variants remained for further analysis (Figure 1). We found n = 87 non-synonymous variants, among which n = 55 variants were related to platelet-associated genes, while n = 32 variants were related to coagulation or fibrinolysis-related genes including the von Willebrand factor gene (*VWF*) and *ADAMTS13*. Most variants were missense mutations (76%) caused by single-nucleotide exchanges at coding regions. All variants were detected in heterozygous form in CTEPH patients, except for a missense variant in *PIGA* (located at chromosome X), which was found in a male patient (c.55C > T, p.Arg19Trp) as hemizygous.

Among genes related to coagulation, variants were found within clotting factor genes *F10*, *F12*, *F13A1*, *F13B, F5* and *F8* (Table 2). Among these, *F10* p.Met336Val, *F12* p.Leu140Val, *F13A1* p.Tyr205Phe, *F13B* intronic mutations and *F5* p.Met1811Leu and p.Met2148Thr were not found in the control group. By investigating genes encoding proteins involved in fibrinolysis, one variant was found in the *KNG1* gene encoding high-molecular-weight kininogen (p.Arg412*), which was absent from the control group. *PLG* (encoding plasminogen) p.Val291Met was also found in one CTEPH patient; however, the plasminogen level was normal in that individual (P12, plasminogen 101 IU/dL) and it is not associated with plasminogen deficiency according to the latest curated databases. *SERPINE1* p.Val17Ile was detected only in one CTEPH patient (P7), whose PAI-1 antigen concentration was below the lower limit of the reference interval (4.1 ng/mL, reference interval 7–43 ng/mL), rather characteristic of a mild PAI-1 deficiency. Among genes encoding proteins serving as natural anticoagulants, an intronic variant in *SERPIND1* encoding heparin cofactor II was found in P2 and it was absent from the control group. We found mutations in the gene encoding thrombomodulin (*THBD*) and in the gene encoding PC (*PROC*), which were also absent from the PE group. The PC level in patient 6 with the mutation c.-21-37G > A in *PROC*, however, was normal (84 IU/dL) and this variant seems not to be causative of PC disorders. Two variants were detected in *ADAMTS13*; one of them (p.Gln1174*) was not found in the control group. Finally, seven different variants were described in *VWF*, among which five mutations were not present in the controls.

Concerning platelet-associated genes, no gene or variant was found, which was potentially relevant from the point of view of CTEPH or any thrombotic phenotype (Supplementary Tables S2 and S3). Genes included in the Tier 1 ISTH database for platelet disorders are rather associated with thrombocytopenia, or platelet function disorders with a bleeding phenotype, mainly if mutations are carried in homozygous form. However, there were some potential exceptions. One CTEPH patient (P10) was a carrier of a *STIM1* mutation (c.1859+1G > A, rs118128831), suggesting a splicing defect. This variant was not found in the control group. The gene is associated with the autosomal dominant Stormorken syndrome with functional asplenia, thrombocytopenia and Howell–Jolly bodies, which are features also described in association with CTEPH. Our patient with a *STIM1* mutation had mild thrombocytopenia with large platelets but he had no Howell–Jolly bodies in his blood smear. A variant of the *THPO* gene (c.889A > G, p.Thr297Ala, rs530613857) was found in another CTEPH patient (P9). An *ETV6* mutation (c.602T > C, p.Leu201Pro, rs145477191) was found in P11. These variants were also absent from the controls.

An additional 11 genes considered as Tier 2 genes according to ISTH recommendation were also investigated. Among them *NFE2, MAST2, APOLD1* and *SERPINA1* were potentially interesting because of their association with clonal hematopoetic regulation, venous thromboembolism, endothelial cell signaling and alpha1-antitrypsin, respectively. By the investigation of rare, non-synonymous variants within these genes, we detected a *SERPINA1* c.863A > T (p.Glu288Val) variant (allele frequency 0.023) in patient P4 and a *NFE2* c.518A > G (p.Asp173Gly) variant (allele frequency unknown) in patient P12. These mutations are considered as likely pathogenic and VUS, respectively, in association with the corresponding diseases according to recent clinical genetic databases. These variants were not detected in the control group.

### 3.5. Investigation of Genes Associated with Vascular Diseases in CTEPH and in PE Patients

Our second virtual gene panel consisted of genes which have been associated with vascular diseases or involved in vascular development, angiogenesis, or thrombotic phenotype, based on literature data and clinical databases (Supplementary Table S4). The following genes were investigated: *ENG*, *ACVRL1*, *BMPR2*, *RASA1*, *GDF2*, *SMAD4*, *SOX17*, *CAV1*, *KCNK3*, *RNF213*, *SMAD9*, *SLC2A10*, *KDR*, *CPB2* and *HRG*. Variant screening was performed for ISTH genes, and variants with MAF above 0.05 and synonymous ones were excluded. By this search, we collected n = 15 different missense or splicing variants (Table 3). Most variants were carried by one patient each, and only *RASA1* p.Ala99Val, *KDR* p.Cys482Arg and *RNF213* p.Leu4283Ile were carried by two CTEPH patients each; however, these mutations were also detected in the control group. There were exclusive variants in *RASA1*, *ENG*, *GDF2*, *SOX17*, *ACVRL1* and *RNF213*, which were not found in PE patients without CTEPH.

Although variants in genes *BMPR2* and *KDR* were also found in PE patients without CTEPH, they might have significance in CTEPH, not directly in its development but rather in its severity and extension, which is well demonstrated by our patients’ clinical histories. All three patients (P1, P4 and P6) had severe and extensive CTEPH, with a total of 13 (out of which only 4 were operable), 14 (out of which only 6 were operable in multiple BPA sessions), and 11 (out of which all were dilated by BPA, but in multiple BPA sessions) segmental pulmonary arteries affected, respectively.

### 3.6. Combination of Panel 1 and 2 Gene Variants in CTEPH Patients

**Figure 2:**
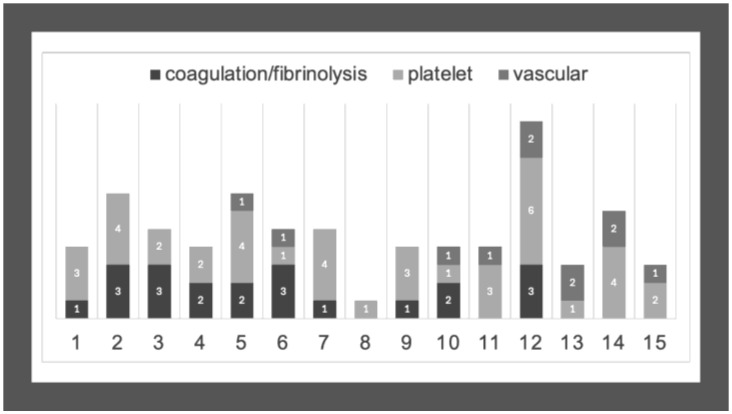
Combined occurrence of variants in CTEPH patients. Numbers on the *X*-axis represent CTEPH patients from P1 to P15. Numbers on the columns represent the number of the detected variants in genes associated with coagulation/fibrinolysis, platelet disorders, or vascular conditions.

We collected the different variants detected in virtual panels 1 and 2 according to CTEPH patients and investigated the combined occurrence of these mutations (Table 4 and Figure 2). The combination of the different variants was rather heterogeneous and in most cases, more than one suspected variant was detected. While there were several variants also found in the control group, we could identify potentially interesting rare variants exclusively detected in CTEPH patients. In most patients, variants related to vascular disorders and those associated with coagulation and fibrinolysis were combined, suggesting the possibility of their additive or synergistic effect.

## 4. Discussion

Despite advances in our understanding of CTEPH genetics, significant knowledge gaps remain regarding the contribution of different genes and variants and the interplay between genetic susceptibility and environmental factors. As expected, no single gene or variant was consequently detected in our CTEPH patients, and no variant was found that could be directly associated with the disease. Our main goal was to investigate rare variants in genes related to thrombosis, hemostasis, and vascular disorders in order to identify potentially interesting ones that are worthy of further study. We compared the presence of the detected variants in CTEPH patients to those in individuals with PE, but without the development of CTEPH. Then we investigated whether the variants found exclusively in the CTEPH group might be associated with the disease, based on clinical genetic databases and/or literature data. It was not surprising that most of the variants were considered as VUS for the corresponding disease in the available databases, and no literature data were available in the context of CTEPH.

Among genes associated with coagulation, only genes encoding different fibrinogen chains and *F5* (due to FV Leiden mutation, p.Arg534Gln) have been associated with CTEPH in previous studies [33,34]. In our cohort, no fibrinogen variants were detected. The FV Leiden mutation was found in one CTEPH patient in our cohort; however, it was also detected in two individuals in the PE (control) group. The presence of FV Leiden was shown to confer a 3-fold risk of early-onset CTEPH in a European study and it represented a risk for CTEPH partly shared with acute PE in a recent large genome-wide association study [35,36]. FV Leiden was not found in idiopathic pulmonary hypertension in that study. Our results support the role of this polymorphism in PE and CTEPH (frequency of FV Leiden is n = 3/32, 10% in the combined group of CTEPH and PE). We have found two other *F5* mutations, p.Met1811Leu and p.Met2148Thr, which were present only in CTEPH patients. While the variant p.Met1811Leu has been reported as a variant of uncertain significance (VUS) for FV-related disorders, p.Met2148Thr has been considered as benign not only from the point of view of bleeding, but also from the point of view of thrombophilia [37].

Mutations in *F10* usually cause FX deficiency and bleeding symptoms mostly in homozygous patients, and no association with thrombotic disorders has been published so far. Therefore, it is not likely that *F10* p.Met336Val, found in CTEPH, may be a risk factor for either CTEPH or thrombosis; however, this variant has not been analyzed in vitro yet. *F12* p.Leu140Val was identified in deep vein thrombosis and also in hereditary angioedema (HAE); however, its pathogenicity has not been clarified in these disorders yet [38] and it seems to be benign in relation to FXII deficiency and severe HAE. However, as coagulation FXII has a role in fibrinolysis and in complement activation rather than in coagulation, this variant may be a candidate for further investigation in a larger cohort of CTEPH patients. Factor XIII is a heterotetrameric molecule with two subunits A and B, where subunit A is the active enzyme transglutaminase and subunit B is a carrier molecule [39]. The involvement of FXIII in thrombotic disorders has been widely investigated with the identification of certain polymorphisms with potential roles in arterial or venous thrombosis. Among them, *F13A1* p.Tyr205Phe, found in one of our patients (P5), has been previously associated with arterial and venous thrombosis, although it was not confirmed as a risk factor for ischemic stroke in a meta-analysis [40]. The patient with this mutation had FXIII activity within the reference interval (131 IU/dL); however, its role in modifying fibrin cross-linking and fibrinolysis cannot be ruled out. Intronic variants in the gene encoding the B subunit of FXIII with unknown allele frequency data were found in a single patient with CTEPH (P3), suggesting a linkage among these alterations. There are still conflicting results of the association of *F13B* polymorphisms with thrombotic diseases; moreover, their association with CTEPH remains unclear [41,42].

The systemic fibrinolysis pathway is typically considered not to be affected in CTEPH, but imbalances in local expression of enzymes involved in fibrinolysis may play a role [22,27]. Among genes associated with fibrinolysis, the *KNG1* variant that leads to a stop codon and suggests the presence of a truncated protein was found in a CTEPH patient and it was absent from controls. This variant (p.Arg412*) was annotated in a large study including patients with venous thrombosis and—since HMWK encoded by this gene is a protein involved in fibrinolysis regulation and in inflammatory processes—it is worthy of further investigation [38]. The *SERPINE1* p.Val17Ile variant may be related to lower secretory dynamics of PAI-1 and to lower PAI-1 levels, as it was also seen in our patient; therefore, the association of this variant with CTEPH is unlikely [43].

Among genes encoding proteins serving as natural anticoagulants, *SERPINC1, PROC* and *PROS1* were investigated in detail earlier, with inconclusive results [44]. In our study, no relevant variants were found in these genes. Instead, an intronic variant was found in *SERPIND1* (c.1309-3C > T), a gene encoding heparin cofactor II, a serin protease inhibitor with a rapid thrombin inhibitory effect in the presence of negatively charged glycosaminoglycans like heparan sulfate, dermatan sulfate, and chondroitin sulfate [45]. Heparin cofactor II seems to be an important factor in the case of atherosclerotic diseases and it seems to prevent vascular restenosis, especially after coronary interventions [46]. Being a natural thrombin inhibitor, whose function is strongly related to vascular wall properties, its role in CTEPH is plausible. It is a question, however, whether this variant has any structural or functional consequences on the protein. This issue is worthy of further investigation, and the role of heparin cofactor II in CTEPH is suggested to be examined in more detail. The *PROC* variant that we found in this study is considered a likely benign mutation according to the clinical databases, which does not influence PC levels, as also seen in our patient. Moreover, it is unlikely that this c.-21-37G > A variant upstream of the coding region of PC would have any effect on the fibrinolysis-regulatory, cytoprotective, and anti-inflammatory functions of PC. The mutation we found in *THBD* (p.Pro501Leu) is an already-described variant with uncertain significance in the context of thrombomodulin-related disorders; however, as thrombomodulin not only regulates thrombus formation but also complement factor I-induced C3b inactivation, its association with CTEPH may be relevant [47,48].

Von Willebrand factor and related proteins have been associated with the development of CTEPH in several studies. Elevated levels of factor VIII and von Willebrand factor were found in patients with CTEPH, suggesting their role in its development; however, this finding also might be a marker of chronic inflammation and endothelial dysfunction in this disease [45]. Although elevated FVIII and vWF levels were described in our CTEPH patients, as compared to controls, no clearly causative mutations for this phenotype were identified in their corresponding genes. There were several *VWF* mutations in our CTEPH patients, among which two variants (p.Arg854Gln and p.Tyr1584Cys) are clearly associated with vWD and bleeding phenotype according to clinical databases, and they are not candidates as CTEPH risk factors [49,50]. The other three mutations (p.Thr1951Ala, p.Arg1399His and p.Thr1054Met) are not considered as vWD-causing ones; however, they may modify vWF levels (or function) and their contribution to thrombotic phenotype cannot be excluded [51]. Our patients carrying these variants all had rather elevated vWF:Ag and vWF:Ac levels. There is no data about *ADAMTS13* p.Gln1174Term mutations in the available databases. It potentially leads to a truncated protein, and may be associated with a thrombotic phenotype with the risk of multiple microvascular thrombus formation.

ISTH-recommended platelet-related genes are basically not relevant from the point of view of CTEPH, as they are mainly associated with bleeding phenotype with thrombocytopenia and/or platelet functional defects. Based on the results of our study, only four platelet-related genes are worthy of consideration. The *STIM1* gene associated with autosomal-dominant Stormorken syndrome with functional asplenia may be a candidate for further studies [52]. *ETV6* encodes a transcriptional repressor, and its variants may be associated with impaired hematopoiesis and clonal abnormalities, whose features were suggested as risk factors for CTEPH [53]. *THPO* variants might be associated with increased thrombopoietin level; thus, recurrent thrombotic episodes may be present in carriers [54]. Finally, variants of the *MPL* gene are associated with thrombocytosis and an abnormal function of thrombopoietin receptor, which makes this gene and its variants considerable factors to be investigated [55].

Vascular genes, especially those involved in hereditary hemorrhagic telangiectasia (HHT) and primary pulmonary hypertension, have been suggested to serve as risk factors for CTEPH development in previous studies [18,56]. We found variants in *ENG* (p.Gly191Asp, p.Thr5Met and p.Pro131Leu), which encodes endoglin, a component of the transforming growth factor-beta receptor complex, and it is an important vascular endothelium-associated glycoprotein. Mutations in *ENG* are mainly responsible for HHT1 [57] and the abovementioned variants are considered as benign from the point of view of this disease. We do not know whether they have any effect on CTEPH development yet. *ACVRL1* c.1378-216C > T is a likely benign mutation in the context of HHT2, and *GDF2* p.Val211Met is a VUS in association with HHT5; however, their effect on CTEPH is not known. *RASA1* is responsible for capillary malformation–arteriovenous malformation syndrome, a phenotype closely related to HHT (OMIM 608354). p.Gly89Arg, which was found in one of our CTEPH patients, is an undescribed variant and is potentially worthy of investigation. We described three variants in *RNF213*, the ring finger protein 213-encoding gene (c.2656-5A > G, p.Thr4638 and p.Gln2184Arg), which were not detected in the controls. As this gene was also suggested to be associated with CTEPH and a variant p.Arg4810Lys was found in patients with bad prognosis, our variants are also worthy of research [58]. *SOX17* is associated with primary pulmonary hypertension (type 7) and its role in CTEPH may be plausible [59]. Variants p.Ala33Asp and p.Met270Leu are considered as likely benign for PPH type 7; however, their behavior in CTEPH is not known. The *KDR* gene encodes a growth factor receptor tyrosine kinase (VEGFR-2), to which vascular endothelial growth factor (VEGF) binds with high affinity, and it is involved in angiogenesis [60]. High VEGFR-2 expression might be associated with pulmonary hypertension [61]. Therefore, it is plausible that variants in *KDR* might play a role in CTEPH. In our cohort, two patients were carriers of a single variant, p.Cys482Arg; however, this was also detected in two control individuals. The role of genetic variants of bone morphogenic protein type II receptor (*BMPR2*) and angiotensin-converting enzyme (*ACE*) in CTEPH was suggested in previous studies; however, their significance is still controversial [62,63]. We did not find mutations in ACE in our cohort, and the mutation in BMPR2 (p.Ser775Asn) that was detected in one CTEPH patient was also found in a control subject. Based on the clinical findings of our patients carrying the *BMPR2* and *KDR* variants, we hypothesize that these genes might play a role not directly in the development of CTEPH but rather in the progression and severity of the disease.

To summarize the results of our study, based on variant interpretations, the following genes may be candidates for more detailed research from the point of view of CTEPH development: *F12*, *F13A1*, *F13B*, *F5*, *KNG1*, *SERPIND1*, *THBD*, *ADAMTS13*, *VWF*, *STIM1*, *ETV6*, *THPO*, *MPL*, *SERPINA1*, *ENG*, *RASA1*, *ACVRL1*, *GDF2*, *NFE2*, *SOX17* and *RNF213*; among these, some were also suggested previously by others [58,64].

We are aware of the small sample size in our study; however, due to the rarity of the disease, it was not possible to collect a larger number of patients. This small sample size, however, does not allow us to draw firm conclusions on the roles of these variants in this disease. It is known that sample size is critical from the point of view of precision and reliability of research findings. A small sample size may lead to inconclusive results, whereas a too-large sample may detect statistically significant but clinically negligible effects [65]. This is also the case in genetic studies, where a small sample size has low power to detect the effects of rare variants individually and can lead to an overestimation of their significance [66]. With this consideration, we cannot state clearly whether the variants found in our cohort have an impact on CTEPH; we rather give an overview of potential candidates for further investigations.

We could not support some previous findings in association with CTEPH in our cohort. First, we did not find alterations in genes encoding fibrinogen. According to a recent meta-analysis, the fibrinogen alpha p.Thr312Ala (*FGA* rs6050) polymorphism is positively associated with susceptibility to venous thromboembolism and CTEPH [67]. Moreover, five fibrinogen heterozygous gene mutations have been previously discovered in CTEPH compared to healthy controls in *FGA* and *FGB* genes, resulting in a disorganized fibrin structure and fibrinolytic resistance; however, these variants were not detected in our cohort either [68]. We also did not find mutations in *CPB2* (TAFI), and the mutation found in *BMPR2* in a CTEPH patient was also detected in the control group [23,69]. We did not detect the *RNF213* p.Arg4810Lys variant in our patients; however, we identified other, potentially interesting rare mutations within this gene [58].

Our study strengthens the hypothesis of heterogeneous genetic background and multifactorial nature of CTEPH, where not only gene–gene but also gene–environment interactions may contribute. The interaction between genetic variants and inflammatory pathways, for example, is an area of ongoing research, as chronic inflammation has been proposed as a contributing factor to thrombus persistence and vascular remodeling [70]. Moreover, gene–environment interactions involving anticoagulant therapy, lifestyle factors, and comorbidities such as obesity and metabolic syndrome may further modulate disease risk [71]. There may be an interplay between several pathways including thrombosis, altered fibrinolysis, defective angiogenesis, and inflammation, which may be inherited or acquired [27]. Recent studies utilizing WES have revealed novel genetic associations with PE and CTEPH, highlighting the potential for precision medicine approaches in identifying at-risk individuals [72,73]. Based on these studies, it is also suggested that a complex interaction between thrombotic–fibrinolytic processes, vascular remodeling, and pulmonary vasculature lesions may exist behind the pathogenesis of CTEPH, in which the contribution of different factors has not been elucidated yet.

These complex interactions are difficult to identify, and clinical studies focusing on distinct areas, like most genetic studies, do not have the chance to shed light on them. Different factors may have only minor effects on the development of the disease, and it is almost impossible to collect all pieces of the puzzle. We can not draw a clear conclusion, but rather may have suggestions for further investigations including larger clinical studies and in vitro experiments to confirm the contribution of a certain factor to the disease.

At our university center, most patients with acute PE are managed and followed using NOACs, reflecting this guideline-driven shift. However, in patients diagnosed with CTEPH, the anticoagulation regimen is typically converted to vitamin K antagonists (VKAs) after diagnosis, as VKAs remain the standard of care supported by stronger evidence in this specific population. These patients are followed by the experts of the CTEPH team. This practice is also endorsed by current CTEPH management recommendations [74]. Lifelong anticoagulation is indicated for all patients with confirmed CTEPH, and this approach was uniformly applied in our study.

Our study has several limitations. First, the sample size is rather small; however, as CTEPH is a rare disease, it is hard to collect a large number of patients in a single center. We focused only on rare variants in genes involved in thrombosis and hemostasis and in vascular diseases and did not investigate others involved in potentially relevant mechanisms, like inflammation, hematopoiesis, or intracellular signaling. We did not have the chance to examine the role of epigenetic factors, like the role of microRNAs in CTEPH or gene expression profile, or factors of clonal hematopoiesis; however, some studies have already described a potentially causative nature of these [75,76,77,78]. We could not study gene–dose effects—which would have been strenghtened genotype–phenotype association—in case of the identified variants, because all patients were heterozygous carriers of the identified mutations. We did not perform in silico analysis of the detected variants in this study; we only used the built-in prediction tools in the software that we applied for NGS data analysis. We did not have the chance to recruit a validation cohort and perform the validation in an independent sample. Despite limitations, our study has the advantage of comparing CTEPH individuals with those with PE but without CTEPH and our results may serve as a starting point for more detailed investigations of certain genes and variants with potential association with CTEPH in larger cohorts.

## 5. Conclusions

By investigation of rare mutations in genes involved in thrombosis and hemostasis and in vascular diseases, several potential candidate variants were identified, whose roles in CTEPH are worthy of further studies. We could not identify a single variant with a higher frequency in our small cohort, and most of the patients carried more than one mutation, suggesting the complex genetic background of the disease. Without further pieces of evidence, their role in CTEPH, of course, remains an assumption.

## Figures and Tables

**Table 1:** Demographic and laboratory characteristics of CTEPH patients and PE patients without the development of CTEPH.

	Patients with CTEPH n = 15	PE Patients Without CTEPH, n = 17	*p* Values
Age at diagnosis of CTEPH/PE (years, mean ± SD)	61.9 ± 15.2	48.9 ± 17.1	0.028
Age at the time of investigation (years, mean ± SD)	63.5 ± 15.7	53.0 ± 16.8	0.079
Gender (n, male/female)	10/5	6/11	0.077
Anticoagulant drug (n, VKA vs. NOAC)	11/4	0/14 *	<0.001
BMI (kg/m^2^, mean ± SD)	27.0 ± 4.2	27.5 ± 6.1	0.806
Blood group (n, O vs. non-O)	3/12	2/15	0.522
Inherited thrombophilia (n, Yes vs. No)	2/13	5/12	0.272
APS (n, Yes vs. No)	2/13	1/16	0.471
FVIII activity (IU/dL, median, range)	170 (102–250)	75 (41–190)	<0.001
vWF:Ag (IU/dL, mean ± SD)	178 ± 47	127 ± 35	0.002
Fibrinogen (g/L, mean ± SD)	3.72 ± 0.54	3.91 ± 0.61	0.398
D-dimer (mg/L, median, range)	0.40 (0.14–4.04)	0.39 (0.19–2.49)	0.970
TAT (μg/L, median, range)	1.6 (0.7–3.4)	2.5 (1.2–60.0)	0.006
PF1+2 (pmol/L, median, range)	93.9 (30.1–221.4)	194.7 (109.4–1200.0)	<0.001
Plasminogen (IU/dL, mean ± SD)	77.7 ± 12.5	102.4 ± 21.6	<0.001
alpha2-PI (IU/dL, median, range)	90.0 (61.0–104.0)	111.0 (71.0–120.0)	<0.001
tPA (ng/mL, median, range)	2.42 (1.38–5.13)	1.40 (0.6–8.31)	0.003
PAI-1 antigen (ng/mL, median, range)	32.3 (4.1–128.1)	30.2 (11.0–130.3)	0.794
FXIII (IU/dL, mean ± SD)	120.5 ± 28.7	134.0 ± 25.4	0.173
TFPI (ng/mL, mean ± SD)	22.23 ± 11.55	25.19 ± 9.14	0.437

VKA, vitamin K antagonist; NOAC, novel oral anticoagulant including rivaroxaban, apixaban and dabigatran; BMI, body mass index; APS, antiphospholipid syndrome; FVIII, coagulation factor VIII; vWF: Ag, von Willebrand factor antigen; TAT, thrombin–antithrombin complex; PF1+2, prothrombin fragment 1+2; alpha2-PI, alpha2 plasmin inhibitor; tPA, tissue plasminogen activator; PAI-1, plasminogen activator inhibitor; FXIII, coagulation factor XIII; TFPI, tissue factor pathway inhibitor; * three PE patients were not anticoagulated at the time of investigation. Reference intervals: FVIII activity 60–150 IU/dL; vWF:Ag 50–160 IU/dL; fibrinogen 1.5–4.0 g/L; D-dimer cut-off 0.5 mg/L; TAT 2.0–4.2 μg/L; PF1+2 69–229 pmol/L; plasminogen 70–140 IU/dL; alpha2-PI 80–120 IU/dL; PAI-1 7–43 ng/mL; FXIII 69–143 IU/dL. No established reference interval is available for TFPI and tPA.

**Table 2:** Variants in genes related to coagulation and fibrinolysis (Tier 1 genes of the ISTH, panel 1) in CTEPH patients.

Gene	ID	Variant (c.DNA)	Variant (Protein)	rs ID	MAF	Present in Controls (YES/NO)
ADAMTS13	P2	c.3520C > T	p.(Gln1174*)	ND	ND	NO
ADAMTS13	P14	c.3097G > A	p.(Ala1033Thr)	rs28503257	0.031	YES
F10	P6	c.1006A > G	p.(Met336Val)	rs942622094	<0.001	NO
F12	P2	c.418C > G	p.(Leu140Val)	rs35515200	0.002	NO
F13A1	P1	c.1951_1954delinsATTC	p.(Val651_Glu652delinsIleGln)	ND	ND	YES
F13A1	P5	c.614A > T	p.(Tyr205Phe)	rs3024477	0.0183	NO
F13B	P3	c.265+1_266-1del	NA	ND	ND	NO
F13B	P3	c.451+1_452-1del	NA	ND	ND	NO
F13B	P3	c.628+1_629-1del	NA	ND	ND	NO
F13B	P2	c.1025T > C	p.(Ile342Thr)	rs17514281	0.0071	YES
F5	P4	c.5431A > T	p.(Met1811Leu)	rs138877178	0.0007	NO
F5	P2	c.1601G > A	p.(Arg534Gln*)	rs6025	0.006	YES
F5	P6	c.6443T > C	p.(Met2148Thr)	rs9332701	0.0306	NO
F8	P3	c.5140A > C	p.(Thr1714Pro)	rs782088688	ND	YES
FGG	P11	c. *496A > C	3′UTR	rs187316301	0.0018	YES
KNG1	P2	c.1290C > G	p.(Asp430Glu)	rs5030084	0.0304	YES
KNG1	P2	c.1925G > C	p.(Gly642Ala)	rs5030087	0.033	YES
KNG1	P9	c.1234C > T	p.(Arg412*)	rs76438938	0.0279	NO
PIGA	P3	c.55C > T	p.(Arg19Trp)	rs34422225	0.0301	YES
PLG	P8	c.266G > A	p.(Arg89Lys)	rs143079629	0.0062	YES
PLG	P12	c.871G > A	p.(Val291Met)	rs564003153	0.0004	NO
PROC	P6	c.-21-37G > A	NA	rs371995306	0.0027	NO
SERPIND1	P2	c.1309-3C > T	NA	rs200548385	0.0004	NO
SERPINE1	P7	c.49G > A	p.(Val17Ile)	rs6090	0.0294	NO
THBD	P12	c.1502C > T	p.(Pro501Leu)	rs1800579	0.0018	NO
VWF	P10	c.5851A > G	p.(Thr1951Ala)	rs144072210	0.0007	NO
VWF	P1	c.4196G > A	p.(Arg1399His)	rs1800382	0.0089	NO
VWF	P12	c.4751A > G	p.(Tyr1584Cys)	rs1800386	0.0026	NO
VWF	P5	c.7682T > A	p.(Phe2561Tyr)	rs35335161	0.038	YES
VWF	P10	c.2561G > A	p.(Arg854Gln)	rs41276738	0.0035	NO
VWF	P5	c.3161C > T	p.(Thr1054Met)	rs757834200	<0.001	NO
VWF	11 pts	c.3515G > T	p.(Gly1172Val)	rs1555195293	ND	YES

ID, patient identification data; NA, not applicable; ND, no data; rs6025 (F5 p.Arg534Gln) corresponds to FV Leiden mutation. MAF, minor allele frequency as obtained from GnomAD or 1000 genomes databases. Control group includes patients with PE, in whom CTEPH was not diagnosed.

**Table 3:** Variants in genes related to vascular disorders/conditions (panel 2) in CTEPH patients.

Gene	ID	Variant (c.DNA)	Variant (Protein)	rs ID	MAF	Present in Controls (YES/NO)
*BMPR2*	P1	c.2324G > A	p.(Ser775Asn)	rs2228545	0.024	YES
*RASA1*	P5, P14	c.296C > T	p.(Ala99Val)	rs111840875	0.0187	YES
*RASA1*	P10	c.265G > A	p.(Gly89Arg	ND	ND	NO
*ENG*	P5	c.572G > A	p.(Gly191Asp)	rs41322046	0.0091	NO
*ENG*	P12	c.392C > T	p.(Pro131Leu)	rs139398993	0.0042	NO
*ENG*	P14	c.14C > T	p.(Thr5Met)	rs35400405	0.0239	NO
*GDF2*	P12	c.631G > A	p.(Val211Met)	rs782438683	<0.001	NO
*SOX17*	P13	c.98C > A	p.(Ala33Asp)	rs189384157	0.0035	NO
*SOX17*	P14	c.807_808delinsAT	p.(Met270Leu)	rs1563871910	ND	NO
*ACVRL1*	P11	c.1378-216C > T	NA	rs111710113	0.0117	NO
*KDR*	P4, P6	c.1444T > C	p.(Cys482Arg)	rs34231037	0.009	YES
*RNF213*	P3, P15	c.12847C > A	p.(Leu4283Ile)	rs62077764	0.0489	YES
*RNF213*	P6	c.2656-5A > G	NA	rs201832175	0.0009	NO
*RNF213*	P13	c.13913C > T	p.(Thr4638Ile)	rs141301945	0.0007	NO
*RNF213*	P15	c.6551A > G	p.(Gln2184Arg)	rs138595111	0.0025	NO

ID, patient identification data; MAF, minor allele frequency as obtained from GnomAD or 1000 genomes databases. Control group includes patients with PE, in whom CTEPH was not diagnosed. *RNF213* c.9848T > C (p.(Leu3283Pro), MAF 0.0001, rs747426139 was found only in control C3. *RNF213* p.Arg4810Lys was not detected in CTEPH or control group either.

**Table 4:** Combination of gene variants with plausible association with CTEPH and/or thrombotic phenotype in Tier 1 and 2 ISTH genes and panel 2 genes in CTEPH patients.

ID	Gene	Variant (c.DNA)	Variant (Protein)	rs ID
P1	**VWF+**	**c.4196G > A**	**p.(Arg1399His)**	**rs1800382**
	*F13A1*	*c.1951_1954delinsATTC*	*p.(Val651_Glu652delinsIleGln)*	*ND*
	*BMPR2*	*c.2324G > A*	*p.(Ser775Asn)*	*rs2228545*
P2	**ADAMTS13+**	**c.3520C > T**	p.(Gln1174* **)**	**ND**
	**F12+**	**c.418C > G**	**p.(Leu140Val)**	**rs35515200**
	*F13B*	*c.1025T > C*	*p.(Ile342Thr)*	*rs17514281*
	*F5*	*c.1601G > A*	*p.(Arg534Gln)*	*rs6025*
	*KNG1*	*c.1290C > G*	*p.(Asp430Glu)*	*rs5030084*
	*KNG1*	*c.1925G > C*	*p.(Gly642Ala)*	*rs5030087*
	**SERPIND1+**	**c.1309-3C > T**	**NA**	**rs200548385**
P3	**F13B**	**c.265+1_266-1del**	**NA**	**ND**
	**F13B**	**c.451+1_452-1del**	**NA**	**ND**
	**F13B**	**c.628+1_629-1del**	**NA**	**ND**
	*F8*	*c.5140A > C*	*p.(Thr1714Pro)*	*rs782088688*
	*PIGA*	*c.55C > T*	*p.(Arg19Trp)*	*rs34422225*
	*RNF213*	*c.12847C > A*	*p.Leu4283Ile*	*rs62077764*
P4	**F5+**	**c.5431A > T**	**p.(Met1811Leu)**	**rs138877178**
	**SERPINA1+**	**c.863A > T**	**(p.Glu288Val**	**rs17580**
	*KDR*	*c.1444T > C*	*p.(Cys482Arg)*	*rs34231037*
P5	**F13A1+**	**c.614A > T**	**p.(Tyr205Phe)**	**rs3024477**
	*VWF*	*c.7682T > A*	*p.(Phe2561Tyr)*	*rs35335161*
	**VWF+**	**c.3161C > T**	**p.(Thr1054Met)**	**rs757834200**
	*RASA1*	*c.296C > T*	*p.(Ala99Val)*	*rs111840875*
	**ENG+**	**c.572G > A**	**p.(Gly191Asp)**	**rs41322046**
P6	**F10**	**c.1006A > G**	**p.(Met336Val)**	**rs942622094**
	**PROC**	**c.-21-37G > A**	**NA**	**rs371995306**
	**RNF213+**	**c.2656-5A > G**	**NA**	**rs201832175**
	*KDR*	*c.1444T > C*	*p.(Cys482Arg)*	*rs34231037*
P7	**SERPINE1**	**c.49G > A**	**p.(Val17Ile)**	**rs6090**
P8	*PLG*	*c.266G > A*	*p.(Arg89Lys)*	*rs143079629*
P9	**KNG1+**	**c.1234C > T**	p.(Arg412 *)	**rs76438938**
	**THPO+**	**c.889A > G**	**p.(Thr297Ala)**	**rs530613857**
P10	**VWF+**	**c.5851A > G**	**p.(Thr1951Ala)**	**rs144072210**
	**STIM1+**	**c.1859+1G > A**	**NA**	**rs118128831**
	**RASA1+**	**c.265G > A**	**p.(Gly89Arg)**	**ND**
P11	*FGG*	c. *496A > C	3′*UTR*	*rs187316301*
	**ETV6+**	**c.602T > C**	**p.(Leu201Pro)**	**rs145477191**
	**ACVRL1+**	**c.1378-216C > T**	**NA**	**rs111710113**
P12	**PLG**	**c.871G > A**	**p.(Val291Met)**	**rs564003153**
	**THBD+**	**c.1502C > T**	**p.(Pro501Leu)**	**rs1800579**
	**ENG+**	**c.392C > T**	**p.(Pro131Leu)**	**rs139398993**
	**GDF2+**	**c.631G > A**	**p.(Val211Met)**	**rs782438683**
	**NFE2+**	**c.518A > G**	**(p.Asp173Gly)**	**ND**
P13	**MPL+**	**c.1666G > T**	**p.(Val556Phe)**	**rs150004498**
	**SOX17+**	**c.98C > A**	**p.(Ala33Asp)**	**rs189384157**
	**RNF213+**	**c.13913C > T**	**p.(Thr4638Ile)**	**rs141301945**
P14	*ADAMTS13*	*c.3097G > A*	*p.(Ala1033Thr)*	*rs28503257*
	**ENG+**	**c.14C > T**	**p.(Thr5Met)**	**rs35400405**
	*RASA1*	*c.296C > T*	*p.(Ala99Val)*	*rs111840875*
	**SOX17+**	**c.807_808delinsAT**	**p.(Met270Leu)**	**rs1563871910**
P15	**RNF213+**	**c.6551A > G**	**p.(Gln2184Arg)**	**rs138595111**

Genes and variants written in **bold** represent mutations that were exclusively found in CTEPH patients. Genes and variants written in *italics* represent mutations that were also found in PE patients without CTEPH (control group). Genes (variants) indicated with a “+” are worthy of further investigation in the context of CTEPH (explanation is in the text). ND, not determined; NA, not applicable; 3′ UTR, 3′ untranslated region. Laboratory phenotype of patients with the corresponding gene variants: P1 has an elevated vWF level (vWF:Ag 262 IU/dL, vWF:Ac 195 IU/dL) and elevated factor VIII (250 IU/dL. FXIII activity was not elevated (97 IU/dL). In P2, rs6025 corresponds to FV Leiden, and FXIII activity was normal (84 IU/dL). FXIII activity was also normal in P3 (100 IU/dL) and his FVIII level was 162 IU/dL. FXIII activity in P5 was 133 IU/dL, and his vWF:Ag was 156 IU/dL. Protein C activity of P6 was normal: 98 IU/dL. PAI-1 level of P7 was 4.1 ng/mL, which corresponds to a mild PAI-1 deficiency. Plasminogen of P8 was normal: 88 IU/dL. vWF:Ag concentration in P10 was 133 IU/dL. The plasminogen level of P12 was 101 IU/dL.

## Data Availability

The original contributions presented in this study are included in the article/Supplementary Material. Further inquiries can be directed to the corresponding author(s).

## Supplementary Materials

The following supporting information can be downloaded at https://www.mdpi.com/article/10.3390/genes16111336/s1. Table S1: Established thrombotic risk factors and/or abnormal values in parameters related to thrombosis in CTEPH patients and in PE patients without the development of CTEPH; Table S2: Variants related to platelet defects and detected in CTEPH patients; Table S3: Tier 1 platelet-dependent genes according to the ISTH recommendation, in which variants were found in CTEPH patients; Table S4: Genes associated with vascular diseases and/or development and included in virtual gene panel 2; Table S5: Combination of variants in Tier 1 and 2 ISTH genes and panel 2 genes in our CTEPH patients.

## Abbreviations

The following abbreviations are used in this manuscript:

6MWT	6-Minute Walk Test
ACMG	American College of Medical Genetics and Genomics
ACE	Angiotensin-Converting Enzyme
ACVRL1	Activin A Receptor-Like Type 1
ADAMTS13	A Disintegrin And Metalloproteinase with Thrombospondin Motifs 13
APTT	Activated Partial Thromboplastin Time
AT	Antithrombin
BMI	Body Mass Index
BMPR2	Bone Morphogenetic Protein Receptor Type 2
BPA	Balloon Pulmonary Angioplasty
CAV1	Caveolin 1
CI	Cardiac Index
CTEPH	Chronic Thromboembolic Pulmonary Hypertension
CTPA	Computed Tomography Pulmonary Angiography
CO	Cardiac Output
CPB2	Carboxypeptidase B2
CT	Computed Tomography
DVT	Deep Vein Thrombosis
DNA	Deoxyribonucleic Acid
ELISA	Enzyme-Linked Immunosorbent Assay
ENG	Endoglin
ETV6	Variant Transcription Factor 6
F10	Coagulation Factor X
F12	Coagulation Factor XII
F13A1	Coagulation Factor XIII A Chain
F13B	Coagulation Factor XIII B Chain
F5	Coagulation Factor V
F8	Coagulation Factor VIII
FGA	Fibrinogen Alpha Chain
FGB	Fibrinogen Beta Chain
FGG	Fibrinogen Gamma Chain
FVIII	Factor VIII
FXIII	Factor XIII
GDF2	Growth Differentiation Factor 2 (also known as BMP9)
GnomAD	Genome Aggregation Database
HAE	Hereditary Angioedema
HGMD	Human Gene Mutation Database
HRG	Histidine-Rich Glycoprotein
HMWK	High-Molecular-Weight Kininogen
IBM SPSS	International Business Machines—Statistical Package for the Social Sciences
IgG	Immunoglobulin G
IgM	Immunoglobulin M
ISTH	International Society on Thrombosis and Haemostasis
KCNK3	Potassium Channel Subfamily K Member 3
KNG1	Kininogen 1
LA	Lupus Anticoagulant
mPAP	Mean Pulmonary Artery Pressure
MAF	Minor Allele Frequency
MAST2	Microtubule-Associated Serine/Threonine Kinase 2
MPL	Myeloproliferative Leukemia Protein
MR	Magnetic Resonance
NFE2	Nuclear Factor, Erythroid 2
NGS	Next-Generation Sequencing
NT-proBNP	N-terminal pro–B-type Natriuretic Peptide
NYHA	New York Heart Association (Functional Classification)
OMIM	Online Mendelian Inheritance in Man
PAI-1	Plasminogen Activator Inhibitor Type 1
PC	Protein C
PCR	Polymerase Chain Reaction
PCWP	Pulmonary Capillary Wedge Pressure
PE	Pulmonary Embolism
PF1+2	Prothrombin Fragment 1+2
PLG	Plasminogen
PROC	Protein C Gene
PROS1	Protein S Gene
PS	Protein S
PVR	Pulmonary Vascular Resistance
RAP	Right Atrial Pressure
RASA1	RAS p21 Protein Activator 1
RNF213	Ring Finger Protein 213
RBBB	Right Bundle Branch Block
SD	Standard Deviation
SERPINA1	Serpin Family A Member 1 (Alpha-1 Antitrypsin)
SERPINC1	Serpin Family C Member 1 (Antithrombin)
SERPIND1	Serpin Family D Member 1 (Heparin Cofactor II)
SERPINE1	Serpin Family E Member 1 (Plasminogen Activator Inhibitor Type 1)
SLC2A10	Solute Carrier Family 2 Member 10
SMAD4	Mothers Against Decapentaplegic Homolog 4
SMAD9	Mothers Against Decapentaplegic Homolog 9
SOX17	SRY-Box Transcription Factor 17
STIM1	Stromal Interaction Molecule 1
TFPI	Tissue Factor Pathway Inhibitor
THBD	Thrombomodulin
THPO	Thrombopoietin
TAT	Thrombin–Antithrombin Complex
tPA	Tissue-Type Plasminogen Activator
V/Q	Ventilation/Perfusion (Scan)
vWF or VWF	von Willebrand Factor
vWF:Ag	von Willebrand Factor Antigen
VUS	Variant of Uncertain Significance
WES	Whole-Exome Sequencing
