# Supplementary material for: Genetic Analysis of Patients with Chronic Thromboembolic Pulmonary Hypertension (CTEPH): A Single-Center Observational Study
Source: Genes (Basel). 2025 Nov 6;16(11):1336. doi: 10.3390/genes16111336 (PMC12652659; doi:10.3390/genes16111336)
Supplement: Supplementary file 1 [file genes-16-01336-s001.zip › genes-3947445-supplementary.pdf]

Supplementary Table S1. Established thrombotic risk factors and/or abnormal values in parameters related to thrombosis in CTEPH patients and in PE patients without the development of CTEPH.

Patient ID	Blood group	Inherited thrombophilia	APS	Elevated FVIII/vWF:Ag	Elevated TAT/PF1+2	Elevated D-dimer	Elevated BMI
P1	non-O	No	No	YES	No	YES	YES
P2	non-O	FVLHez	No	No	No	No	No
P3	non-O	PT20210AHoZ	No	No	No	YES	No
P4	non-O	No	No	No	No	YES	No
P5	O	No	anti-B2GPI IgG	No	No	No	YES
P6	non-O	No	No	No	No	YES	YES
P7	non-O	No	No	No	No	No	No
P8	non-O	No	No	YES	No	No	YES
P9	O	No	LA+	YES	No	YES	No
P10	non-O	No	No	No	No	No	No
P11	non-O	No	No	No	No	YES	No
P12	non-O	No	No	No	No	No	No
P13	non-O	No	No	No	No	No	No
P14	non-O	No	No	YES	No	No	No
P15	O	No	No	YES	No	YES	No
C1	non-O	No	No	No	No	No	No
C2	non-O	No	No	No	YES	No	YES
C3	O	No	No	No	No	YES	No
C4	non-O	No	No	No	No	YES	No
C5	non-O	FVLHez	No	No	No	No	No
C6	O	PT20210AHeZ	No	No	No	No	No
C7	non-O	No	No	No	YES	No	YES
C8	non-O	No	No	No	YES	YES	No
C9	non-O	No	No	No	No	No	No
C10	non-O	No	No	No	YES	No	No
C11	non-O	No	No	No	No	No	YES
C12	non-O	FVLHez	No	No	YES	YES	No
C13	non-O	No	No	No	YES	No	YES
C14	non-O	No	No	No	No	No	No
C15	non-O	PS Heerlen HeZ	No	No	YES	YES	No
C16	non-O	No	No	No	No	No	No
C17	non-O	PT20210AHeZ	LA+	YES	No	YES	No

FVLHeZ, Factor V Leiden heterozygous; PT20210AHeZ, prothrombin gene 20210A allele heterozygous; PT20210AHoZ, prothrombin gene 20210A allele homozygous; PS Heerlen HeZ, PROS1 c.1501T>C, p.Ser501Pro heterozygous; LA+, lupus anticoagulant positive; anti-B2GPI IgG, medium titer elevation in anti-beta2 glycoprotein I IgG isotype antibody; APS, antiphospholipid syndrome; FVIII, factor FVIII level; vWF:Ag, von Willebrand factor antigen; TAT, thrombin-antithrombin complex; PF1+2, prothrombin fragment 1+2; BMI, body mass index, elevated BMI was considered above 30kg/m^2.

Supplementary Table S2. Variants related to platelet defects and detected in CTEPH patients.

gene	patient ID	variant (c.DNA)	variant (protein)	rs ID	MAF	present also in PE patients (YES/NO)
ABCC4	P14	c.3425C>T	p.Thr1142Met	rs11568644	0.0067	YES
ABCC4	P6	c.559G>T	p.Gly187Trp	rs11568658	0.0464	YES
ABCC4	P4	c.2560G>T	p.Val854Phe	rs11568694	0.0072	NO
ABCG5	P14	c.1567A>G	p.Ile523Val	rs140899003	0.0018	NO
ABCG5	P7	c.593G>A	p.Arg198Gln	rs141828689	0.0013	NO
ABCG8	P11	c.722C>T	p.Ser241Phe	rs547583131	0.0003	NO
ANO6	P2	c.529C>G	p.Pro177Ala	rs753570988	ND	NO
BLOC1S3	P6	c.92C>T	p.Ala31Val	rs148910210	0.0001	NO
BLOC1S3	P14	c.478G>T	p.Val160Leu	rs201502372	0.0017	YES
BLOC1S3	P7	c.322C>G	p.Leu108Val	rs75792246	0.0266	YES
DIAPH1	P2	c.145-90T>C	NA	rs55662777	0.0393	NO
DTNBP1	P3	c.811+78C>T	NA	rs16876571	0.0115	YES
DTNBP1	P1	c.811+59A>G	NA	rs16876573	0.0348	YES
DTNBP1	P8	c.814C>T	p.Pro272Ser	rs17470454	0.0437	YES
ETV6	P11	c.602T>C	p.Leu201Pro	rs145477191	0.005	NO
FLNA	P8	c.4451A>G	p.Gln1484Arg	rs200130356	0.001	NO
GP1BA	P12	c.1369A>G	p.Thr457Ala	rs1302217377	0	NO
GP6	P8	c.1495G>A	p.Gly499Ser	rs41275822	0.0245	YES
HPS1	P7, P11	c.847G>T	p.Gly283Trp	rs11592273	0.0491	YES
HPS1	P6	c.11T>C	p.Val4Ala	rs58548334	0.0322	YES
HPS3	P11	c.2692C>T	p.Arg898Cys	rs543058717	0.0006	YES
HPS3	P14	c.2637C>A	p.Phe879Leu	rs774685745	0	NO
HPS4	P11	c.670-44G>A	NA	rs372772133	0.0002	NO
HPS4	P15	c.1720C>T	p.Leu574Phe	rs9625029	0.008	NO
HPS5	P8, P10	c.3293C>T	p.Thr1098Ile	rs61884288	0.0237	YES
ITGA2B	P2	c.439C>G	p.Leu147Val	rs76066357	0.0091	NO
LYST	P9	c.5518T>G	p.Ser1840Ala	rs115330112	0.0044	NO
LYST	P12	c.3931A>G	p.Met1311Val	rs376718077	0.0001	NO
MECOM	P1	c.884A>G	p.Gln295Arg	rs34896995	0.0023	NO
MPL	P13	c.1666G>T	p.Val556Phe	rs150004498	0.0002	NO
MPL	P9	c.1565+5C>T	NA	rs41269541	0.0046	NO
MPL	P3, P5	c.1058A>G	p.His353Arg	rs756953598	0	YES
MYH9	P15	c.998A>G	p.Glu333Gly	ND	ND	NO
MYH9	P7	c.5323A>G	p.Lys1775Glu	rs145139708	0.0016	NO
MYH9	P2	c.136C>T	p.Leu46Phe	rs147122501	0.0062	NO
MYH9	P5	c.5483+4C>G	NA	rs56327920	0.0159	NO
NBEA	P1	c.7039A>G	p.Ile2347Val	rs189755961	0.0048	NO
NBEA	P5, P13	c.4504A>G	p.Ser1502Gly	rs41292197	0.0346	YES
NBEAL2	P14	c.5660C>T	p.Pro1887Leu	rs202203116	0.0001	NO
NBEAL2	P14	c.386C>T	p.Thr129Met	rs372831354	0.0001	NO
NBEAL2	P12	c.5745G>C	p.Glu1915Asp	rs771627544	0	NO

PLA2G4A	P5, P12	c.1909A>G	p.Ile637Val	rs28395831	0.0084	NO
RUNX1	P7	c.98-21T>C	NA	rs371813424	0.0002	NO
STIM1	P10	c.1859+1G>A	p.(?)	rs118128831	0.0074	NO
STXBP2	P7	c.1034C>T	p.Thr345Met	rs117761837	0.0106	NO
STXBP2	P3, P5	c.795-4C>T	NA	rs151257815	0.0117	NO
TBXAS1	P4, P1	c.236+22T>G	NA	rs17161199	0.0123	NO
TBXAS1	P15	c.1349C>A	p.Thr450Asn	rs5763	0.0141	YES
TBXAS1	P6	c.480C>A	p.Asp160Glu	rs5768	ND	YES
TBXAS1	P3	c.1345G>A	p.Glu449Lys	rs8192868	0.0139	NO
THPO	P9	c.889A>G	p.Thr297Ala	rs530613857	0.0001	NO
VPS33B	P6	c.1166G>A	p.Arg389Gln	rs145070485	0.0021	YES
VPS33B	P5	c.1170+5G>A	NA	rs201431055	0.0009	NO

NA, not applicable; ND, no data; MAF, minor allele frequency as obtained from GnomAD or 1000genomes databases.

Supplementary Table S3. Tier 1 platelet-dependent genes according to the ISTH recommendation, in which variants were found in CTEPH patients.

**gene**	**Associated disorder/phenotype**	**inheritance**
ABCC4	Reduced ADP-induced platelet aggregation	AR
ABCG5	Sitosterolemia with macrothrombocytopenia	AR
ABCG8	Sitosterolemia with macrothrombocytopenia	AR
ANO6	Scott syndrome, reduced calcium-dependent platelet signaling	AR
BLOC1S3	Hermansky-Pudlak syndrome	AR
DIAPH1	Macrothrombocytopenia and sensorineural hearing loss	AD
DTNBP1	Hermansky-Pudlak syndrome	AR
ETV6	Thrombocytopenia and susceptibility to cancer	AD
FLNA	Macrothrombocytopenia	XD/XR
GP1BA	Bernard-Soulier sy., mild macrothrombocytopenia, platelet-type vWD	AR/AD
GP6	Bleeding diathesis due to glycoprotein VI deficiency	AR
HPS1	Hermansky-Pudlak syndrome	AR
HPS3	Hermansky-Pudlak syndrome	AR
HPS4	Hermansky-Pudlak syndrome	AR
HPS5	Hermansky-Pudlak syndrome	AR
ITGA2B	Glanzmann-thrombasthenia, platelet-type bleeding disorder 16	AR/AD
LYST	Chediak-Higashi syndrome	AR
MECOM	Amegakaryocytic thrombocytopenia with radioulnar synostosis 2	AD
MPL	Congenital amegakaryocytic thrombocytopenia (CAMT)	AR
MYH9	May-Hegglin and other MYH9 disorders	AD
NBEA	Platelet dense granule defect	AD
NBEAL2	Gray platelet syndrome	AR
PLA2G4A	Deficiency of phospholipase A2, group IV A	AR
RUNX1	Familial platelet disorder with predisposition to AML	AD
STIM1	Stormorken syndrome	AD
STXBP2	Familial hemophagocytic lymphohistiocytosis type, reduced platelet dense granule secretion	AR
TBXAS1	Thromboxane A synthase defect, Aspirin-like effects in platelet aggregation	AR

THPO	Thrombopoetin deficiency, thrombocytopenia	AR, AD
VPS33B	ARC syndrome (Arthrogryposis, renal dysfunction, and cholestasis 2) with larger platelets, decreased alpha granules and increased dense granules	AR

STIM1-associated Stormorken syndrome usually associates with a short statue, functional asplenia, or splenic aplasia, thrombocytopenia, Howell-Jolly bodies, some of them are features also described in association with CTEPH. Our patient with STIM1 mutation has a short statue, mild thrombocytopenia with large platelets but he has no Howell-Jolly bodies in his blood smear.

Supplementary Table S4. Genes associated with vascular diseases and/or development and included in virtual gene panel 2.

**gene**	**Encoded protein/Associated disorder (phenotype)**	**OMIM accession No.**	**inheritance**
ENG	Endoglin/hereditary hemorrhagic telangiectasia	131195	AD
ACVRL1	Activin A receptor, type II-like kinase 1/ hereditary hemorrhagic telangiectasia, type 2	601284	AD
BMPR2	Bone morphogenetic protein receptor, type II/pulmonary hypertension with or without HHT or pulmonary venoocllusive disease 1	600799	AD
RASA1	RAS p21 protein activator 1/capillary malformation-arteriovenous malformation 1	139150	AD
GDF2	Growth/differentiation factor 2/ hereditary hemorrhagic telangiectasia, type 3	605120	AD
SMAD4	SMAD family member 4/ juvenile polyposis/hereditary hemorrhagic telangiectasia syndrome	600993	AD
SOX17	Sry-box 17/pulmonary hypertension, primary, 7	610928	AD
CAV1	Caveolin 1/pulmonary hypertension, primary, 3	615343	AD
KCNK3	Potassium channel, subfamily K, member 3/pulmonary hypertension, primary, 4	615344	AD
RNF213	Ring finger protein 213/moyamoya disease 2	607151, 613768	AD/AR
SMAD9	SMAD family member 9/pulmonary hypertension, primary, 2	615342, 603295	AD
SLC2A10	Solute carrier family 2/arterial tortuosity sy	208050, 606145	AR
KDR	Kinase insert domain receptor	602089	AD
CPB2	Carboxypeptidase B2 (TAFI)/fibrinolysis alterations	603101	ND
HRG	Histidine-rich glycoprotein/thrombophilia	613116	AD

Genes in virtual panel 2 were recruited based on literature data in the context of vascular diseases, angiogenesis and on previous findings concerning their association with CTEPH (please see in the text for more details).

Supplementary Table S5. Combination of variants in Tier 1 and 2 ISTH genes and panel 2 genes in our CTEPH patients.

**Patient ID**	**gene**	**variant (c.DNA)**	**variant (protein)**	**rs ID**
P1	VWF	c.4196G>A	p.(Arg1399His)	rs1800382
	F13A1	*c.1951_1954delinsATTC*	*p.(Val651_Glu652delinsIleGln)*	*ND*

	NBEA	c.7039A>G	p.(Ile2347Val)	rs189755961
	MECOM	c.884A>G	p.(Gln295Arg)	rs34896995
	TBXAS1	c.236+22T>G	NA	rs17161199
	DTNBP1	*c.811+59A>G*	*NA*	*rs16876573*
	BMPR2	*c.2324G>A*	*p.(Ser775Asn)*	*rs2228545*
P2	ADAMTS13	c.3520C>T	p.(Gln1174*)	ND
	F12	c.418C>G	p.(Leu140Val)	rs35515200
	F13B	*c.1025T>C*	*p.(Ile342Thr)*	*rs17514281*
	F5	*c.1601G>A*	*p.(Arg534Gln)*	*rs6025*
	KNG1	*c.1290C>G*	*p.(Asp430Glu)*	*rs5030084*
	KNG1	*c.1925G>C*	*p.(Gly642Ala)*	*rs5030087*
	SERPIND1	c.1309-3C>T	NA	rs200548385
	ANO6	c.529C>G	p.(Pro177Ala)	rs753570988
	DIAPH1	c.145-90T>C	NA	rs55662777
	ITGA2B	c.439C>G	p.(Leu147Val)	rs76066357
	MYH9	c.136C>T	p.(Leu46Phe)	rs147122501
P3	F13B	c.265+1_266-1del	NA	ND
	F13B	c.451+1_452-1del	NA	ND
	F13B	c.628+1_629-1del	NA	ND
	F8	*c.5140A>C*	*p.(Thr1714Pro)*	*rs782088688*
	DTNBP1	*c.811+78C>T*	*NA*	*rs16876571*
	MPL	*c.1058A>G*	*p.(His353Arg)*	*rs756953598*
	PIGA	*c.55C>T*	*p.(Arg19Trp)*	*rs34422225*
	STXBP2	c.795-4C>T	NA	rs151257815
	TBXAS1	c.1345G>A	p.(Glu449Lys)	rs8192868
	RNF213	*c.12847C>A*	*p.(Leu4283Ile)*	*rs62077764*
P4	F5	c.5431A>T	p.(Met1811Leu)	rs138877178
	ABCC4	*c.559G>T*	*p.(Gly187Trp)*	*rs11568658*
	ABCC4	c.2560G>T	p.(Val854Phe)	rs11568694
	TBXAS1	c.236+22T>G	NA	rs17161199
	SERPINA1	c.863A>T	p.(Glu288Val)	rs17580
	KDR	*c.1444T>C*	*p.(Cys482Arg)*	*rs34231037*
P5	F13A1	c.614A>T	p.(Tyr205Phe)	rs3024477
	VWF	*c.7682T>A*	*p.(Phe2561Tyr)*	*rs35335161*
	VWF	c.3161C>T	p.(Thr1054Met)	rs757834200
	MPL	*c.1058A>G*	*p.(His353Arg)*	*rs756953598*
	MYH9	c.5483+4C>G	NA	rs56327920
	NBEA	*c.4504A>G*	*p.(Ser1502Gly)*	*rs41292197*
	PLA2G4A	c.1909A>G	p.(Ile637Val)	rs28395831
	STXBP2	c.795-4C>T	NA	rs151257815

	VPS33B	c.1170+5G>A		rs201431055
	RASA1	*c.296C>T*	*p.(Ala99Val)*	*rs111840875*
	ENG	c.572G>A	p.(Gly191Asp)	rs41322046
P6	F10	c.1006A>G	p.(Met336Val)	rs942622094
	F5	c.6443T>C	p.(Met2148Thr)	rs9332701
	PROC	c.-21-37G>A	NA	rs371995306
	BLOC1S3	c.92C>T	p.(Ala31Val)	rs148910210
	HPS1	*c.11T>C*	*p.(Val4Ala)*	*rs58548334*
	TBXAS1	*c.480C>A*	*p.(Asp160Glu)*	*rs5768*
	VPS33B	*c.1166G>A*	*p.(Arg389Gln)*	*rs145070485*
	RNF213	c.2656-5A>G	NA	rs201832175
	KDR	*c.1444T>C*	*p.(Cys482Arg)*	*rs34231037*
P7	SERPINE1	c.49G>A	p.(Val17Ile)	rs6090
	ABCG5	c.593G>A	p.(Arg198Gln)	rs141828689
	BLOC1S3	*c.322C>G*	*p.(Leu108Val)*	*rs75792246*
	HPS1	*c.847G>T*	*p.(Gly283Trp)*	*rs11592273*
	MYH9	c.5323A>G	p.(Lys1775Glu)	rs145139708
	RUNX1	c.98-21T>C	NA	rs371813424
	STXBP2	c.1034C>T	p.(Thr345Met)	rs117761837
P8	*PLG*	*c.266G>A*	*p.(Arg89Lys)*	*rs143079629*
	DTNBP1	*c.814C>T*	*p.(Pro272Ser)*	*rs17470454*
	FLNA	c.4451A>G	p.(Gln1484Arg)	rs200130356
	GP6	*c.1495G>A*	*p.(Gly499Ser)*	*rs41275822*
	HPS5	*c.3293C>T*	*p.(Thr1098Ile)*	*rs61884288*
P9	KNG1	c.1234C>T	p.(Arg412*)	rs76438938
	LYST	c.5518T>G	p.(Ser1840Ala)	rs115330112
	MPL	c.1565+5C>T	NA	rs41269541
	THPO	c.889A>G	p.(Thr297Ala)	rs530613857
P10	VWF	c.5851A>G	p.(Thr1951Ala)	rs144072210
	VWF	c.2561G>A	p.(Arg854Gln)	rs41276738
	HPS5	*c.3293C>T*	*p.(Thr1098Ile)*	*rs61884288*
	STIM1	c.1859+1G>A	NA	rs118128831
	RASA1	c.265G>A	p.(Gly89Arg)	ND
P11	*FGG*	*c.*496A>C*	*3′UTR*	*rs187316301*
	ABCG8	c.722C>T	p.(Ser241Phe)	rs547583131
	ETV6	c.602T>C	p.(Leu201Pro)	rs145477191
	HPS1	*c.847G>T*	*p.(Gly283Trp)*	*rs11592273*
	HPS3	*c.2692C>T*	*p.(Arg898Cys)*	*rs543058717*
	HPS4	c.670-44G>A	NA	rs372772133
	ACVRL1	c.1378-216C>T	NA	rs111710113
P12	PLG	c.871G>A	p.(Val291Met)	rs564003153
	THBD	c.1502C>T	p.(Pro501Leu)	rs1800579
	VWF	c.4751A>G	p.(Tyr1584Cys)	rs1800386

	GP1BA	c.1369A>G	p.(Thr457Ala)	rs1302217377
	LYST	c.3931A>G	p.(Met1311Val)	rs376718077
	NBEAL2	c.5745G>C	p.(Glu1915Asp)	rs771627544
	PLA2G4A	c.1909A>G	p.(Ile637Val)	rs28395831
	TRPM7	c.458A>G	p.(Lys153Arg)	rs2061163602
	ENG	c.392C>T	p.(Pro131Leu)	rs139398993
	GDF2	c.631G>A	p.(Val211Met)	rs782438683
	NFE2	c.518A>G	(p.Asp173Gly)	ND
P13	MPL	c.1666G>T	p.(Val556Phe)	rs150004498
	NBEA	*c.4504A>G*	*p.(Ser1502Gly)*	*rs41292197*
	SOX17	c.98C>A	p.(Ala33Asp)	rs189384157
	RNF213	c.13913C>T	p.(Thr4638Ile)	rs141301945
P14	*ADAMTS13*	*c.3097G>A*	*p.(Ala1033Thr)*	*rs28503257*
	ABCC4	*c.3425C>T*	*p.(Thr1142Met)*	*rs11568644*
	ABCG5	c.1567A>G	p.(Ile523Val)	rs140899003
	BLOC1S3	*c.478G>T*	*p.(Val160Leu)*	*rs201502372*
	HPS3	c.2637C>A	p.(Phe879Leu)	rs774685745
	NBEAL2	c.5660C>T	p.(Pro1887Leu)	rs202203116
	NBEAL2	c.386C>T	p.(Thr129Met)	rs372831354
	TRPM7	*c.5309-5T>C*	*NA*	*rs117899712*
	ENG	c.14C>T	p.(Thr5Met)	rs35400405
	RASA1	*c.296C>T*	*p.(Ala99Val)*	*rs111840875*
	SOX17	c.807_808delinsAT	p.(Met270Leu)	rs1563871910
P15	HPS4	c.1720C>T	p.(Leu574Phe)	rs9625029
	MYH9	c.998A>G	p.(Glu333Gly)	ND
	TBXAS1	*c.1349C>A*	*p.(Thr450Asn)*	*rs5763*
	RNF213	c.6551A>G	p.(Gln2184Arg)	rs138595111

Genes and variants in *Italics* indicate, that they are also found in the control group (ie. PE patients without the development of CTEPH). Genes and variants with the regular style indicate, that they are only present in CTEPH patients. ND, not determined; NA, non applicable
